# Retro-Curve Access Sheath for Retroverted Anatomy in Left Atrial Appendage Closure: Bench Validation and Patient-Specific Simulation Study

**DOI:** 10.1016/j.shj.2026.100820

**Published:** 2026-02-19

**Authors:** Vlad Ciobotaru, Meyer Elbaz, Xavier Iriart, Frederic Bouisset, Guillaume Bonnet, Victor-Xavier Tadros, Emmanuel Teiger, Julien Ternacle, Sébastien Hascoët

**Affiliations:** a Cardiology Department, Hôpital Privé les Franciscaines, Nîmes, France; b Cardiac Imaging Department, Radiology Division, CHU Nîmes – Hôpital Carémeau, Nîmes, France; c Hôpital Marie Lannelongue, Hôpitaux Paris-Saint-Joseph et Marie Lannelongue, Faculté de Médecine Kremlin-Bicêtre, Université Paris-Saclay, Inserm UMR-S 999, MALIC, Le Plessis-Robinson, France; d 3DHeartModeling, Nîmes, France; e Department of Cardiology, Rangueil University Hospital, Toulouse, France; f Bordeaux University Hospital, Institut Hospitalo-Universitaire Liryc, Electrophysiology and Heart Modeling Institute, Fondation Bordeaux Université, Bordeaux, France; g Department of Cardiology, APHM, La Timone University Hospital, Hôpital Privé La Casamance, Marseille, France; h Department of Cardiology, AP-HP, Henri Mondor University Hospital, Créteil, France

**Keywords:** 3D printing, Bench testing, Coaxiality, Left atrial appendage closure, Patient-specific simulation, Retrocurve access sheath design, Retroverted left atrial appendage

## Abstract

• Left atrial appendage (LAA)-Print registry analysis identified 22 retroverted LAA anatomies treated with WATCHMAN.
• Retro LAA was associated with high rates of off-axis deployment, leak, and failure.
• All 22 cases underwent bench testing using soft patient-specific atrial models.
• Double-curve and steerable sheaths remained noncoaxial despite puncture optimization.
• A newly designed retro-curve sheath restored axial alignment regardless of puncture site.

Misalignment between the delivery sheath and the left atrial appendage (LAA) axis is a major determinant of off-axis deployment, device instability, and peridevice leak during LAA closure, particularly in retroverted anatomies where posterior LAA orientation constrains coaxial engagement despite transseptal puncture optimization.^1^

To address this limitation, a retro-curve delivery sheath was developed from the clinically approved WATCHMAN delivery system. The design combines a primary curvature for redirection from the transseptal puncture with a secondary posterior curvature engineered to restore coaxiality in retroverted anatomies. This geometry facilitates torque-free tracking of the native LAA axis where standard double-curve sheaths remain anteriorly constrained. Prototype shaping was performed in collaboration with Transluminal (France), with intellectual property pending (3DHeartModeling, France).

The performance of this sheath geometry was evaluated in all retroverted LAA anatomies from the LAA-Print Registry (NCT03330210) with WATCHMAN implantations (22/117 cases, 19%). Retroversion was defined as an angle (Φ) >180° between the proximal LAA axis and the mitral annular plane. Reversed chicken-wing morphology, defined by marked proximal angulation, was present in 16/22 cases (73%).

Compared with nonretroverted anatomy, retroverted LAA was associated with higher rates of implantation failure (9/22: 41% vs. 6/117: 5%), off-axis deployment (100% vs. 9%), and peridevice leak (18/22: 82% vs. 10/117: 9%) (all *p* < 0.0001). Implantation failure tended to be more frequent in reversed chicken-wing anatomies (8/16) than in nonangulated retroverted configurations (2/6).

To enable controlled and reproducible assessment, all testing was performed using soft, highly deformable thermoplastic polyurethane patient-specific 3D-printed atrial models reproducing deformation under torque.^2^ Standard double-curve and retro-curve sheaths were tested under identical inferoposterior (PI) and anteroinferior (AI) trans-septal puncture sites, accessed through a transparent membrane bonded to each patient-specific atrial model to simulate the interatrial septum and ensure a standardized puncture location across all bench-tested models.

Within this framework, coaxiality assessment integrated both geometric alignment and mechanical effort, with marked deformation considered a negative criterion. Alignment was assessed by comparing the delivery sheath trajectory with the proximal LAA axis and was graded as favorable, borderline, or unfavorable. Favorable alignment included direct coaxial access or mild obliquity (<30°) correctable with light torque; borderline alignment reflected deviations >30°; unfavorable alignment corresponded to tangential or divergent trajectories requiring excessive torque and deformation. Bench findings were correlated with clinical WATCHMAN implantation outcomes on postprocedural computed tomography.

Accordingly, the primary endpoint was alignment performance. Technical success was defined as stable deployment without excessive torque or model deformation. Device fit was assessed by off-axis orientation, peridevice gap, and bulging.

With the standard double-curve sheath, PI access resulted in 100% unfavorable alignment. AI puncture partially improved alignment, with 45.5% borderline cases, but no favorable alignment and 54.5% remaining unfavorable. In contrast, the retro-curve sheath achieved favorable alignment in 95.5% of cases with AI access and 68.2% with PI access, with limited borderline (18.2%) and unfavorable (13.6%) alignment, indicating relative independence from puncture location.

Consistent with alignment findings, the retro-curve sheath restored successful deployment in cases previously associated with in situ failure, achieving torque-free implantation in 21 of 22 cases (95.5%; *p* = 0.003), without peridevice gap or off-axis deployment in all but 1 case (*p* < 0.0001 vs. double-curve) (Figure 1).

**Figure 1:**
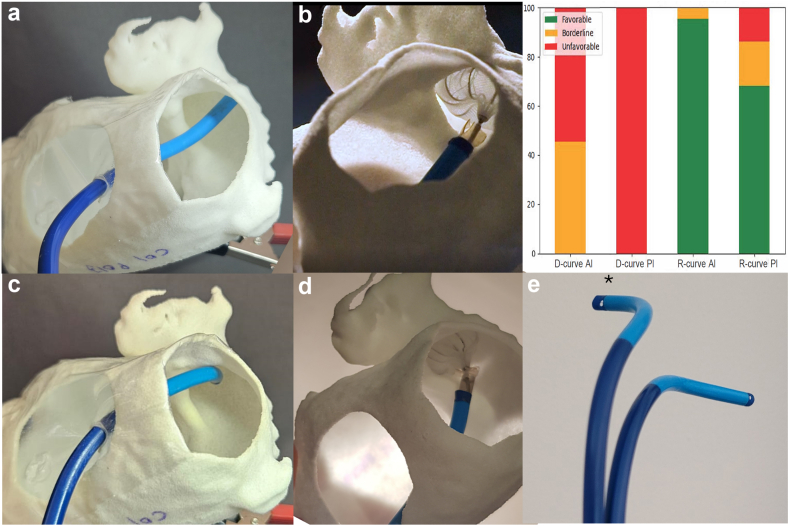
Impact of sheath geometry on coaxiality and device deployment in retroverted LAA **(a)** Anterior oblique view showing marked misalignment between a standard double-curve sheath and the proximal axis of a retroverted left atrial appendage (LAA) after an antero-inferior transseptal puncture. **(b)** Resulting off-axis WATCHMAN deployment with a visible peridevice gap. **(c)** Restoration of axial alignment using the retro-curve delivery sheath through the same transseptal puncture site. **(d)** In-axis WATCHMAN deployment with complete ostial coverage and absence of peridevice leak. **(e)** Lateral comparison of double-curve and retro-curve sheath (∗) geometries highlighting posterior redirection with the retro-curve design. Graph. Distribution of alignment grades across the retroverted LAA cohort (n = 22), according to sheath type (double-curve vs. retro-curve) and transseptal puncture site (anteroinferior [AI] and posteroinferior [PI]). Favorable (green), borderline (orange), and unfavorable (red) alignment grades.

As a complementary analysis, bench testing of a steerable double-curve sheath in 5 randomly selected models showed no favorable alignment. AI access yielded borderline alignment in 3/5 cases and unfavorable alignment in the remaining cases, whereas PI access remained predominantly unfavorable (4/5), indicating limited correction of coaxiality in retroverted anatomy (Supplemental Figure).

Regarding interobserver agreement, 1 interobserver discordance was observed with the double-curve sheath (1/22). For the retro-curve sheath, interobserver discordance occurred in 4 cases with PI access and 3 with AI access; for graphical representation, the most unfavorable category was retained.

Taken together, retroverted LAA represents a distinct access-related anatomical challenge in LAA closure.^3^ Although AI trans-septal puncture may partially improve alignment compared with PI access,^4^ residual misalignment frequently persists in markedly retroverted configurations. Steerable delivery systems may improve catheter verticality but do not generate posterior curvature; in bench testing, coaxiality remained highly dependent on puncture location and was not consistently restored. In contrast, the fixed retro-curve sheath intrinsically restores posterior orientation, enabling coaxial access with reduced torque and procedural complexity.

This bench-testing analysis is based on a limited number of retroverted LAA cases. However, derivation from the LAA-Print Registry allowed direct correlation between bench findings and real-life outcomes. These results should be considered hypothesis-generating and warrant validation in larger multicenter studies.

## Ethics Statement

The study was approved by the local ethics committee, and all patients provided written informed consent.

## Funding

The authors have no funding to report.

## Disclosure Statement

Vlad Ciobotaru is the founder and CEO of 3DHeartModeling. Julien Ternacle reports consulting fees from 10.13039/100004313General Electric, Philips Healthcare, Abbott Structural, 10.13039/100006520Edwards Lifesciences, Pi-Cardia, and TRiCares.

The other authors had no conflicts to declare.

## Figures and Tables

**Supplemental Figure:**
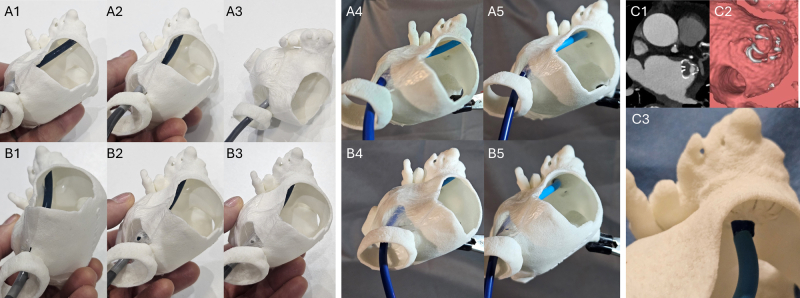
Bench testing in a retroverted LAA anatomy. Panels A1–A5 (anteroinferior transseptal puncture). In the neutral configuration, the WATCHMAN TruSteer delivery sheath reproduces a standard double-curve geometry, with the catheter trajectory remaining parallel to the LAA ostium and resulting in unfavorable axial alignment (A1). Upward deflection combined with marked clockwise torque induces partial catheter verticalization but fails to restore true coaxiality with the proximal LAA axis (A2); panel A3 confirms the anteroinferior puncture site. Introduction of a fixed double-curve sheath through the same puncture site maintains divergent and unfavorable alignment (A4). In contrast, the retro-curve delivery sheath restores axial alignment and enables coaxial engagement of the posterior proximal LAA (A5). Panels B1–B5 (posteroinferior transseptal puncture). With the WATCHMAN TruSteer delivery sheath, marked lack of coaxiality persists despite both upward and downward deflection maneuvers (B1–B3), and a similar unfavorable alignment is observed with the fixed double-curve sheath (B4). By comparison, the retro-curve sheath achieves coaxial alignment with the LAA ostium and proximal posterior LAA axis without applied torque (B5). Panels C1–C2. In situ WATCHMAN deployment showing significant bulging, peridevice leak (PDL), and marked off-axis positioning secondary to delivery sheath misalignment. Panel C3. Optimal prosthesis seating and coaxial deployment achieved during bench simulation using the retro-curve delivery sheath. Abbreviations: LAA, left atrial appendage.

## References

[1] Freixa X., Tzikas A., Basmadjian A., Garceau P., Ibrahim R. (2013). The chicken-wing morphology: an anatomical challenge for left atrial appendage occlusion. J Interv Cardiol.

[2] Ciobotaru V., Batistella M., Emmer E.D.O. (2024). Aortic valve engineering advancements: precision tuning with laser sintering additive manufacturing of TPU/TPE submillimeter membranes. Polymers.

[3] Yang M., Chen M., Gong C.-Q. (2023). Left atrial appendage closure in patients with reversed chicken-wing morphology: anatomical features and procedural strategy. Heliyon.

[4] Fukutomi M., Fuchs A., Bieliauskas G. (2022). Computed tomography-based selection of transseptal puncture site for percutaneous left atrial appendage closure. EuroIntervention.

